# Study of non-Hodgkin's lymphoma mortality associated with industrial pollution in Spain, using Poisson models

**DOI:** 10.1186/1471-2458-9-26

**Published:** 2009-01-21

**Authors:** Rebeca Ramis, Enrique Vidal, Javier García-Pérez, Virginia Lope, Nuria Aragonés, Beatriz Pérez-Gómez, Marina Pollán, Gonzalo López-Abente

**Affiliations:** 1 Department of Environmental Epidemiology and Cancer, National Centre for Epidemiology, Carlos III Institute of Health, Madrid, Spain; 2 Consortium for Biomedical Research in Epidemiology & Public Health (*CIBER en Epidemiología y Salud Pública *– *CIBERESP*), Madrid, Spain

## Abstract

**Background:**

Non-Hodgkin's lymphomas (NHLs) have been linked to proximity to industrial areas, but evidence regarding the health risk posed by residence near pollutant industries is very limited. The European Pollutant Emission Register (EPER) is a public register that furnishes valuable information on industries that release pollutants to air and water, along with their geographical location.

This study sought to explore the relationship between NHL mortality in small areas in Spain and environmental exposure to pollutant emissions from EPER-registered industries, using three Poisson-regression-based mathematical models.

**Methods:**

Observed cases were drawn from mortality registries in Spain for the period 1994–2003. Industries were grouped into the following sectors: energy; metal; mineral; organic chemicals; waste; paper; food; and use of solvents. Populations having an industry within a radius of 1, 1.5, or 2 kilometres from the municipal centroid were deemed to be exposed. Municipalities outside those radii were considered as reference populations.

The relative risks (RRs) associated with proximity to pollutant industries were estimated using the following methods: Poisson Regression; mixed Poisson model with random provincial effect; and spatial autoregressive modelling (BYM model).

**Results:**

Only proximity of paper industries to population centres (>2 km) could be associated with a greater risk of NHL mortality (mixed model: RR:1.24, 95% CI:1.09–1.42; BYM model: RR:1.21, 95% CI:1.01–1.45; Poisson model: RR:1.16, 95% CI:1.06–1.27). Spatial models yielded higher estimates.

**Conclusion:**

The reported association between exposure to air pollution from the paper, pulp and board industry and NHL mortality is independent of the model used. Inclusion of spatial random effects terms in the risk estimate improves the study of associations between environmental exposures and mortality.

The EPER could be of great utility when studying the effects of industrial pollution on the health of the population.

## Background

In general, industrial activities constantly release a great amount of toxic substances into the environment. At present, evidence regarding the health risk posed by residing near pollutant industries and, by extension, being exposed to their emissions, is limited. Non-Hodgkin's lymphomas (NHLs) constitute one of the tumour sites that has been linked in the literature to proximity to industrial areas [1-3]. During the second half of the 20^th ^century, NHLs witnessed a marked increase world-wide, in terms of both incidence and mortality [4], which means that they form part of the group of so-called emerging tumours. This same increase has also been observed in Spain [5].

Although this tumour's aetiology is rather unknown, its relationship with the immune system has generated theories about its increase being connected with the HIV epidemic [6], though the inclusion of Highly Active Antiretroviral Treatments (HAARTs) does not appear to have affected the rising trend in NHLs [7].

From the environmental point of view, there are some studies that link lymphomas to exposure to substances such as agricultural chemicals [7], and dioxins released by incinerators [8]. Mention should also be made of the fact that a number of occupational exposure studies have reported higher NHL incidence and mortality among workers exposed to industrial solvents [6,9,10]. According to Spanish mortality data, NHLs are particularly frequent in the Canary Islands [11], while on the mainland, higher NHL mortality is observed in Asturias, the Basque Country and Catalonia, three of Spain's most industrialised regions [12].

At the beginning of this decade, specific legislation was passed, both in Spain and in Europe, governing the control of pollutant emissions. This initiative included the setting-up of the public European Pollutant Emission Register (EPER) [13], which records pollutant emissions reported by industries that admit to exceeding pre-established pollution thresholds included in the Decision (2000/479/EC) of the European Commission [14]. This database furnishes information on the location of industrial foci, 50 specific pollutants, and a long list of industrial processes that release emissions to air and water, thereby offering a wide range of possibilities for using such information to study possible associations between risk of incidence or mortality due to different causes and proximity to sources of industrial emissions.

Quality information on industrial pollution, as part of the overall environmental pollution to which the population is exposed, is a critical point when it comes to evaluating its effects. Due to the dearth of such information, a recourse widely used in scientific literature is to estimate exposure based on the distance to the polluting source [1-3,15].

This study sought to explore the relationship between municipal NHL mortality in Spain and distance to EPER-registered industries, as an indirect measure of exposure to industrial pollution, using a series of Poisson-regression-based mathematical models for the purpose.

## Methods

Observed NHL cases, broken down by death, sex and age group (18 groups), were drawn from entries of individual deaths recorded by the National Statistics Institute (*Instituto Nacional de Estadística *– *INE*) with ICD9 for the period 1994–2003, in respect of the 8073 Spanish towns registered in the 2001 census.

Municipal populations, likewise broken down by sex and age group, were used to calculate expected cases These populations were obtained from the 1996 electoral roll and the 2001 census, which respectively correspond to the mid-point of the two five-year periods included in the study (1994–1998 and 1999–2003). Person-years for each quinquennium were calculated by multiplying the respective populations by 5. Expected cases resulted from multiplying the mortality rates for Spain as a whole, for each sex, age group and quinquennium, by the person-years of each town, broken down by the same strata.

Industrial pollution data were obtained from the EPER figures published in 2004, which include industries that voluntarily reported pollutant emissions exceeding a designated reporting threshold for 50 toxic substances. This database contains information identifying the industrial activity, the substances emitted, and the installation's geographical location by reference to its co-ordinates, previously validated and corrected for poor geocoding [16]. The emission data correspond to information reported by industries for 2001. The 452 industries that reported releases to air to the EPER were grouped by industrial sector. In this study, farms were excluded from the analysis.

For the construction of the exposure variable and calculation of RRs on the basis of spatial autocorrelation models, maps of municipal boundaries and co-ordinates of the centroids of population centres were used. This is the only available geographical information for each municipality, boundaries of the township and its centroid. We do not know the real limits of the inhabited areas; consequently, we assume that the whole population of each town lives in its centroid.

Distance to the emission source was used as an estimator of exposure to pollutant substances released by industries, [15,17-19]. Using this criterion, exposed populations were defined as any population corresponding to a town that had EPER-registered industries situated within a radius of 2000, 1500 and 1000 metres, in a circle drawn with the municipal centroid as its centre. For study purposes, we only considered industrial groups that had a minimum of 10 towns within the 2-, 1.5- and 1-kilometre areas respectively. On the basis of this definition, an exposure variable was constructed for each industrial sector that showed more than 10 towns having industries within the predefined radius, for the total Spanish population. This variable was defined as a factor with three possible levels, which distinguished among: towns that had no industrial installation within the designated radius (unexposed); towns that had installations corresponding to the industrial group studied within the designated radius (exposed); and towns that had some other type of industrial installation. Finally, with the aim of controlling possible confounding effects, the following socio-demographic variables were included in the analysis: percentage of illiteracy; percentage of unemployed persons; size of household (persons per home), obtained from the 1991 census; and mean income level [20].

### Models

Firstly, Poisson regression models were fitted, using the following formula:

$$\begin{array}{l} O_{i}\sim Po\left(\mu_{i}=E_{i}\lambda_{i}\right)\\ \log\left(\lambda_{i}\right)=\sum_{j}\beta_{j}x_{i}\Rightarrow \log\left(\mu_{i}\right)=\log\left(E_{i}\right)+\sum_{j}\beta_{j}x_{i} \end{array}$$

where: λ_i _is the relative risk in area i; O_i _is the number of deaths in area i; E_i _are the expected cases; and x_i _are the socio-demographic variables.

This risk estimation method takes no account of any possible spatial correlation in data drawn from contiguous areas, such as towns in a given region or country. To take such correlation into account, we therefore considered a mixed Poisson-regression-based model that included a provincial random effect:

$$\begin{array}{l} O_{i}\sim Po\left(\mu_{i}=E_{i}\lambda_{i}\right)\\ \log\left(\lambda_{i}\right)=\sum_{j}\beta_{j}x_{i}+p_{i}\Rightarrow \log\left(\mu_{i}\right)=\log\left(E_{i}\right)+\sum_{j}\beta_{j}x_{i}+p_{i} \end{array}$$

where *p*_*i *_is the provincial random term.

Lastly, a Bayesian hierarchical model was used [21-23]. These types of models, which fall within the category of the so-called conditional autoregressive models (CAR), include two random-effects terms that take the following into account: a) municipal contiguity (spatial term); and b) municipal heterogeneity. In our case, we used the model proposed by Besag, York and Molliè (BYM) [24]:

$$\begin{array}{l} O_{i}\sim Po\left(\mu_{i}=E_{i}\lambda_{i}\right)\\ \log\left(\lambda_{i}\right)=\sum_{j}\beta_{j}x_{i}+h_{i}+b_{i}\Rightarrow \log\left(\mu_{i}\right)=\log\left(E_{i}\right)+\sum_{j}\beta_{j}x_{i}+h_{i}+b_{i}\\ \;\begin{array}{l} h_{i}\sim Normal(\mu ,\tau_{h})\\ b_{i}\sim Car.Normal(\eta_{i},\tau_{b})\\ \tau_{h}\sim Gamma(\alpha ,\beta )\\ \tau_{b}\sim Gamma(\gamma ,\delta ) \end{array} \end{array}$$

where: h_i _is the term of municipal heterogeneity; and b_i _is the spatial term.

With each of the three methodologies used, a multivariate model was fitted including the distance to the locus of each type of industry, individually, and the remaining possible confounders mentioned above. In the Poisson and mixed models, the estimates were calculated using the *glm *and *glmmPQL *functions of the R software programme [25]. Spatial autocorrelation models were fitted with the aid of the WinBUGS Bayesian estimation programme [26]. To obtain results from the spatial model, a burn-in period of 150,000 iterations was performed, which guaranteed convergence of the model parameters, and the posterior distribution was derived with a further 25,000 iterations. Approximately 15 hours on a conventional computer was required to complete this process.

## Results

From 1994 and 2003 there were 22,262 NHL-related deaths in Spain, accounting for 2.7% of all cancer deaths. In 4758 towns (59%) there was no death due to this cause.

The industrial sectors considered, together with the number of towns respectively located less than 2000, 1500 and 1000 metres away, are shown in Table 1. This table also includes the population belonging to towns deemed to be exposed within a radius of 2000 metres.

**Table 1:** Industrial sectors.

		**No. towns with installations at x metres**				
	**Industrial sector**	***Total No. factories***	**2000**	**1500**	**1000**	**Population exposed at <2000 m**
1	Combustion installations > 50 MW	*59*	24	13	6	1,034,398
2	Mineral oil and gas refineries	*10*	3	3	1	9,520
3	Metal industry and metal ore roasting or sintering installations, Installations for the production of ferrous and non-ferrous metals	*68*	52	35	22	113,953
4	Installations for the production of cement clinker (>500 t/d), lime (>50 t/d), glass (>20 t/d), mineral substances (>20 t/d) or ceramic products (>75 t/d)	*55*	79	49	27	665,785
5	Basic organic chemicals	*37*	18	8	3	284,852
6	Basic inorganic chemicals or fertilisers	*25*	5	3	1	169,963
7	Pharmaceutical products	*8*	4	2	1	216,590
8	Installations for the disposal or recovery of hazardous waste (>10 t/d) or municipal waste (>3 t/h)	*8*	4	0	0	62,853
9	Installations for the disposal of nonhazardous waste (>50 t/d) and landfills (>10 t/d)	*43*	15	4	1	146,811
10	Industrial plants for pulp from timber or other fibrous materials and paper or board production (>20 t/d)	*18*	13	6	3	725,225
11	Slaughterhouses (>50 t/d), plants for the production of milk (>200 t/d), other animal raw materials (>75 t/d) or vegetable raw materials (>300 t/d)	*12*	14	9	4	208,227
12	Installations for surface treatment or products using organic solvents (>200 t/y)	*12*	13	6	2	418,749

Number of towns with installations at distances of 2000, 1500, 1000 and 500 metres from the municipal centroid, by type of industry. Population exposed to emissions from each industrial sector at a distance of 2000 metres.

Shown in Table 2 are the RRs associated with each of the industrial sectors studied, for the respective radii of 2000, 1500 and 1000 metres. This table also includes the confidence (models 1 and 2) and credibility intervals (model 3) of the estimates. From these estimates, it will be seen that in towns situated within a radius of 2000 metres of paper, pulp and board installations, exposure to pollutant emissions from this industry was associated with excess NHL mortality. This excess risk was statistically significant in all 3 models, namely: 1.163 (95% CI: 1.06,1.27) for Poisson regression; 1.24 (95% CI: 1.09,1.42) for the mixed model; and 1.21 (95% CI: 1.01,1.45) for the spatial BYM model. Analysing the RRs associated with the variable of exposure to the paper, pulp and board industry in Table 2, it will be seen that the highest RR estimate was yielded by the spatial mixed model, followed by the BYM model and Poisson regression, in that order.

**Table 2:** Relative risks and 95% confidence and credibility intervals for towns with installations lying within a radius of 2000, 1500 and 1000 metres from the municipal centroid.

		**Poisson**			**Mixed**			**BYM**		
**Radius**	**Industrial Sector**	**RR**	**Lower limit**	**Upper limit**	**RR**	**Lower limit**	**Upper limit**	**RR**	**2.50%**	**97.50%**

	Combustion installations > 50 MW	1.06	0.94	1.19	1.08	0.95	1.24	1.10	0.93	1.29
	
	Metal industry and metal ore roasting or sintering installations, Installations for the production of ferrous and non-ferrous metals	0.92	0.85	1.00	0.96	0.87	1.06	0.97	0.85	1.10
	
	Installations for the production of cement clinker (>500 t/d), lime (>50 t/d), glass (>20 t/d), mineral substances (>20 t/d) or ceramic products (>75 t/d)	0.90	0.81	1.01	0.96	0.86	1.08	0.96	0.84	1.10
	
	Basic organic chemicals	1.01	0.87	1.17	1.02	0.88	1.20	1.08	0.89	1.30
	
**2000 m**	Installations for the disposal of non-hazardous waste (>50 t/d) and landfills (>10 t/d)	0.89	0.71	1.11	0.96	0.76	1.21	0.92	0.70	1.19
	
	**Industrial plants for pulp from timber or other fibrous materials and paper or board production (>20 t/d)**	**1.16**	**1.06**	**1.27**	**1.24**	**1.09**	**1.42**	**1.21**	**1.01**	**1.45**
	
	Slaughterhouses (>50 t/d), plants for the production of milk (>200 t/d), other animal raw materials (>75 t/d) or vegetable raw materials (>300 t/d)	0.91	0.75	1.10	0.99	0.81	1.21	0.99	0.77	1.27
	
	Installations for surface treatment or products using organic solvents (>200 t/y)	0.95	0.83	1.10	1.10	0.93	1.31	1.14	0.91	1.43

	Combustion installations > 50 MW	1.11	0.93	1.33	1.09	0.91	1.31	1.07	0.84	1.35
	
**1500 m**	Metal industry and metal ore roasting or sintering installations, Installations for the production of ferrous and non-ferrous metals	0.92	0.81	1.05	0.93	0.81	1.06	0.96	0.82	1.12
	
	Installations for the production of cement clinker (>500 t/d), lime (>50 t/d), glass (>20 t/d), mineral substances (>20 t/d) or ceramic products (>75 t/d)	0.83	0.71	0.98	0.87	0.74	1.03	0.89	0.73	1.07

**1000 m**	Metal industry and metal ore roasting or sintering installations, Installations for the production of ferrous and non-ferrous metals	0.92	0.78	1.08	0.89	0.75	1.05	0.91	0.74	1.10
	
	Installations for the production of cement clinker (>500 t/d), lime (>50 t/d), glass (>20 t/d), mineral substances (>20 t/d) or ceramic products (>75 t/d)	0.97	0.77	1.23	1.00	0.79	1.26	1.03	0.79	1.33

Estimates adjusted for age, sex and socio-demographic variables.

## Discussion

The results show a possible association between exposure to air pollution from the paper, pulp and board industry and excess risk of NHL mortality, regardless of which model is used. Analysing the information contained in the EPER for 2001 shows that almost all the paper, pulp and boardindustries reported emissions of the following compounds, above the threshold established for their inclusion in the registry: CO; CO_2_; NO_2_; sulphur dioxide; organochlorinated compound mixtures; and organic carbon. Taken individually, some of these industries also reported emissions of metals (chrome, copper, nickel, lead and zinc), as well as phosphorous, nitrogen and PM10 particulate matter.

In the literature, there are few studies that link NHL to environmental exposure to chemical substances. Some occupational studies suggest a positive association with exposure to organic solvents, such as benzene [27-29], trichloroethylene (TCE), tetrachloroethylene (PCE) and styrene [29,30]. Other occupational studies associate exposure to pesticides with an elevated risk of NHLs [31,32]. Lastly, different studies addressing the relationship between NHLs and exposure to dioxins furnish contradictory results [33,34]. In one study on a large cohort of paper industry workers, mortality from non-Hodgkin's lymphoma and leukaemia was higher among workers with elevated SO(2) exposure, and a dose-response relationship with cumulative SO(2) exposure was suggested for non-Hodgkin's lymphoma. The cohort included 57,613 workers who had been employed for a minimum of 1 year in the pulp and paper industry in 12 countries [35]. Aside from environmental exposures, there is evidence to indicate that situations associated with chronic antigenic stimulation or immunosupression favour the appearance of these tumours [6,7].

Assessment of exposure to environmental agents that are noxious to human health is a very complex process. At present, there is a great variety of exposure-measurement strategies, depending on the timeliness and availability of resources, which include the use of remote sensors, biomarkers, or estimates of pollutant dispersion using theoretical or statistical models [36]. With respect to this last avenue of research, there are a number of studies in the literature that seek to estimate the risk associated with proximity to hazardous sites (focused clustering) [2,37]. In these and other studies, the authors have explored the idea of estimating risk according to distance [2-4,15].

At present, the real availability of data from remote sensors or biomarkers is negligible. Hence, in the absence of such information, many studies have used distance as an exposure marker. This approach has been further refined, by endeavouring to model pollutant dispersion using anisotropic models that take data, such as wind direction or geographical relief [17], into account. These models could not be applied to this study, however, for lack of information of this type.

With respect to our study, using the distance from the industry to the municipal centroid means that, as the study radius is reduced, the number of towns deemed to be exposed falls drastically. This situation leads to the elimination of exposure variables and the impossibility of studying variation in risk according to a more stringent definition of exposure for most of the emissions considered. Based on the results for the two industrial groups analysed at the three distances (production and processing of metals and mineral industries), no conclusion can be reached as to variation in risk with variation in distance to the emission source.

In ecological spatial correlation studies, Poisson regression is one of the basic tools applied to analysing the association between risk of mortality and the various potential risk factors [15,17]. This type of regression forms part of so-called generalised linear models and assumes independence between observations or counts, an assumption that could be violated when working with data that have a spatial structure [15,21]. Nevertheless, the use of Poisson regression may help obtain an initial assessment of the presence or absence of this association. Indeed, a number of authors have used this method to evaluate the relationship between risk factors and excess incidence or mortality in the study of non-communicable diseases in a spatial context [37,38]. The second model used -the mixed model- is included as an intermediate step between a model that assumes total independence and a model that assumes autocorrelation among observations, and has the advantage of circumventing the problems of extra-Poisson dispersion, lending robustness to the estimators and using the provincial level to approach autocorrelation, which amounts to a form of stratification in the comparisons. Lastly, the third model -the BYM model- assumes that each observation is conditionally independent of the others, i.e., that observations are spatially correlated amongst themselves, with the aim of modelling the spatial effect of the risk [24,38,39]. In none of the models, multiple comparison adjustment was considered. The probability of one spurious test result was 0.33. Due to this low probability and the number of comparisons, we decided to asses the adjustment for multiple testing by the consistency of the associations showed by the results of the different models.

In our results for almost all the industrial sectors considered, the related risks were observed to increase as the random effects covered by the spatial structure of the data were included. The relative risks yielded by the mixed model are, in general, higher than those yielded by the Poisson regression, while those yielded by the BYM model are the highest for most of the variables. The inclusion of random spatial effects terms in risk estimation, not only improves the study of the associations between environmental exposures and mortality, but also reduces proneness to "ecological bias" as a result of working on a larger scale and adjusting for unknown confounders which have a spatial distribution different to that of mortality [21]. However, bearing the similarity of results in mind, the decision to apply the spatial model in exploratory studies of this magnitude must be carefully evaluated, due to the excessive time of computation. The ever increasing availability of health and exposure data calls for the definition of a fast and easy methodology of analysis that would optimise available resources within research groups when it came to embarking upon exploratory studies [23].

None of the socio-demographic variables considered in our study appeared to act as a potential confounder, inasmuch as their elimination in the various models led to no substantial changes in the effect estimators of the distance to the industrial foci studied (data not shown). Furthermore, these possible confounding variables, defined *a priori*, displayed no important direct effect on risk of NHL mortality, registering RRs close to unity.

As stated above, little is known about the possible role of environmental exposures in NHL aetiology, which may be due to the fact most of the studies undertaken to date focused on small towns and poor-quality exposure measures. This implies a limited statistical power that hinders the estimate of modest RRs [8]. This paper presents a first approach to the exploration of the influence of exposures to industrial air pollution and risk of NHL mortality vis-à-vis the entire population of a country, something that is an advantage in terms of the sheer size of the exposed population but is a drawback in terms of possible misclassification of exposure or the uniqueness of each of the installations.

Other possible limitation is the use of ICD9, that classification has not different code for each type of lymphoma included in the LNH; as a result we can not know the spatial patterns of each individual type. Moreover, mortality data only includes the more aggressive type of lymphoma. Less aggressive lymphomas have a low mortality rate and, consequently, they are not included in this study.

It should also be pointed out that the data referring to environmental industrial exposures were drawn from the first edition of the EPER. The quality of this information may conceivably improve with the new European Pollutant Release and Transfer Register (E-PRTR), which will completely replace the EPER in 2009, thereby allowing for the validity of a study of this type to be enhanced, with the possibility of evaluating the effect of specific pollutants. Moreover, though the "near versus far" analysis conducted in this study assumes all the industries of a single sector to be equal, it must nevertheless be borne in mind that each industrial source has its own characteristics, and subsequent studies will therefore have to address these on a case-by-case basis.

Finally, we should not forget that the use of aggregated data implies important assumptions. We assume that the whole population within a municipality lives in its centroid; even more, we assume that they have always been living there. Also, we do not consider the daily movement of the people to go to work or study, for instance. Hence, we are assuming that everybody within an area is exposed to the same type and amount of pollutant substances.

## Conclusion

The results suggest a possible increased risk of NHL mortality among populations residing in the vicinity of paper and pulp industries, an excess mortality that is observable using different models. In order to confirm or reject these results, it would be of great interest to seek to improve the exposure markers and ascertain precisely what is happening in the environs of each specific installation. In addition, the availability of incidence data would be very useful to study less aggressive lymphomas with low mortality rate, which are not included in this study. Those data would provide valuable information to analyse the spatial patterns of individual type of lymphomas integrated in modern classifications of the LNH in reference to specific locations and exposures. Unfortunately, currently there are no incidence data available at national level in Spain.

## Abbreviations

NHLs: Non-Hodgkin's lymphomas; EPER: European Pollutant Emission Register; RR: relative risk;

## Competing interests

The authors declare that they have no competing interests.

## Authors' contributions

RR and GLA conceived the idea. RR performed the statistical analysis and wrote the first draft of the manuscript to which all authors subsequently contributed. GLA, MP, NA, and BPG were all involved in designing the study. EV, JGP and GLA reviewed the statistical analysis and the information about possible pollutant sources in the areas with high relative risk. All authors made contribution to interpretation of results, revised the manuscript for important intellectual content and read and approved the final manuscript.

## Pre-publication history

The pre-publication history for this paper can be accessed here:


